# Wheat genotypes differing in aluminum tolerance differ in their growth response to CO_2_ enrichment in acid soils

**DOI:** 10.1002/ece3.559

**Published:** 2013-04-15

**Authors:** Qiuying Tian, Xinxin Zhang, Yan Gao, Wenming Bai, Feng Ge, Yibing Ma, Wen-Hao Zhang

**Affiliations:** 1 State Key Laboratory of Vegetation and Environmental Change, Institute of Botany, Chinese Academy of SciencesBeijing, 100093, China; 2 State Key Laboratory of Integrated Management of Pest Insects and Rodents, Institute of Zoology, Chinese Academy of SciencesBeijing, China; 3 Research Network of Global Change Biology, Beijing Institutes of Life Science, The Chinese Academy of SciencesBeijing, China; 4 National Soil Fertility and Fertilizer Effects Long-term Monitoring Network, Institute of Agricultural Resources and Regional Planning, Chinese Academy of Agricultural SciencesBeijing, 100081, PR China

**Keywords:** Acid soils, Aluminum tolerance, elevated CO_2_, growth response, malate efflux, wheat (*Triticum aestivum* L.)

## Abstract

Aluminum (Al) toxicity is a major factor limiting plant growth in acid soils. Elevated atmospheric CO_2_ [CO_2_] enhances plant growth. However, there is no report on the effect of elevated [CO_2_] on growth of plant genotypes differing in Al tolerance grown in acid soils. We investigated the effect of short-term elevated [CO_2_] on growth of Al-tolerant (ET8) and Al-sensitive (ES8) wheat plants and malate exudation from root apices by growing them in acid soils under ambient [CO_2_] and elevated [CO_2_] using open-top chambers. Exposure of ET8 plants to elevated [CO_2_] enhanced root biomass only. In contrast, shoot biomass of ES8 was enhanced by elevated [CO_2_]. Given that exudation of malate to detoxify apoplastic Al is a mechanism for Al tolerance in wheat plants, ET8 plants exuded greater amounts of malate from root apices than ES8 plants under both ambient and elevated [CO_2_]. These results indicate that elevated [CO_2_] has no effect on malate exudation in both ET8 and ES8 plants. These novel findings have important implications for our understanding how plants respond to elevated [CO_2_] grown in unfavorable edaphic conditions in general and in acid soils in particular.

## Introduction

The atmospheric concentration of CO_2_ ([CO_2_]) in the Earth's atmosphere has increased from about 280 to 390 ppm since 1800, and it is predicted to reach up to 970 ppm by the end of the 21st century (IPCC, 2007). Elevated [CO_2_] can enhance the rate of photosynthesis of leaves due to higher CO_2_ concentrations at the sites where CO_2_ is fixed by enzymes, and reduce water loss due to partial closure of stomata, thus leading to stimulation of plant growth. In general, elevated [CO_2_] can enhance growth of C3 plants by up to 59% (Kirschbaum 2011). The elevated [CO_2_]-induced enhancement of photosynthesis and growth is diminished gradually over time, a phenomenon known as CO_2_ acclimation (Long et al. 2004). There have been numerous studies investigating the effect of elevated [CO_2_] on plant growth at different scales in the literature (Poorter and Pérez-Soba 2001; Korner 2006). The stimulatory effect of elevated [CO_2_] can also depend on other environmental factors such as soil N (Reich et al. 2006) and water status, salinity, low temperature, ozone (Poorter and Pérez-Soba 2001), and UV-B radiation (Bussell et al. 2012). In addition to nitrogen, recent studies also revealed that growth response of plants to elevated [CO_2_] can also depend on availability of other mineral nutrients (see review of Lenka and Lal 2012), including phosphorus (Wasaki et al. 2005; Sicher 2009; Bussell et al. 2012; Jin et al. 2012), iron (Haase et al. 2008; Jin et al. 2009), and boron (Mishra et al. 2012). It has been generally concluded that plants growing in nutrient-poor conditions is less responsive to elevated [CO_2_] than those growing in nutrient-rich conditions (Poorter 1998; Poorter and Pérez-Soba 2001; Lenka and Lal 2012). It has been suggested that mineral stress resulting from either deficiencies in mineral nutrients and/or toxicity of excessive minerals may play an important role in our understanding how global climate change will affect plants in real world soils (Lynch and St.Clair 2004; Akhter et al. 2009). However, the majority of research on the effect of elevated [CO_2_] on plant growth has mainly focused on the interaction between elevated [CO_2_] and the mineral nutrients essential for plant growth and development so far, whereas a few studies have examined the responsiveness of plant growth to [CO_2_] enrichment in the presence of toxic metals in soils. For example, Jia et al. (2010) investigated the effects of elevated [CO_2_] on growth and photosynthetic characteristics in *Lolium mutiforum* and *L. perenne* in the presence of Cd in soils.

Aluminum (Al) is the most abundant metal and the third most abundant chemical element in the Earth's crust. Al is non-toxic to plants and living organisms under neutral and alkaline conditions (May and Nordstrom 1991). However, Al is hydrolyzed to be phytotoxic Al^3+^ cations in acidic soils, which account for ∼30% of the arable land worldwide, and Al toxicity is a major environmental factor limiting plant growth and crop production in acidic soils (Kochian et al. 2005). Soil acidification can result from imbalances in nitrogen, sulfur, and carbon cycles, excess uptake of cations over anions, nitrogen fixation by legumes (Tang and Rengel 2003), and long-term application of N fertilizers (Guo et al. 2010). Inhibition of root elongation is one of the most distinct symptoms in plants suffering from Al toxicity (Rengel and Zhang 2003). Root apex in general and the distal part of the elongation zone in particular are critical sites for perception and expression of Al toxicity and tolerance (Ryan et al. 1993; Sivaguru and Horst 1998; Khan et al. 2009). A number of plant species and genotypes within species exhibit an inheritable resistance to toxic Al^3+^. One important mechanism for tolerance of plants to Al is achieved by exudation of organic anions, including malate, citrate, and oxalate, from root apices (Ma et al. 2001; Ryan et al. 2001; Kochian et al. 2004). The organic anions released from the root apices can detoxify the toxic Al^3+^ cations by forming non-toxic complexes of Al-organic anions in the rhizosphere, thus protecting root apices from Al^3+^ damage. In wheat, tolerance to Al is mainly achieved by exuding malate anions from root apices through activation of malate-permeable channels, thus detoxifying toxic Al in the rhizosphere (Delhaize et al. 1993; Ryan et al. 1995; Zhang et al. 2001). There has been extensive research on the role of Al-dependent malate efflux in wheat tolerance to Al (Ma et al. 2001; Ryan et al. 2001, 2011; Kochian et al. 2004). Given that large amounts of arable soils are becoming acidified worldwide, particularly in China due to inappropriate management of N fertilization (Guo et al. 2010), and that the atmospheric CO_2_ concentrations are continuously rising (IPCC 2007), elucidation of mechanisms underlying the adaptation of plants to the environments with soil acidification and elevated [CO_2_] will be essential for breeding crops that are capable of growing in acid soils under elevated [CO_2_]. In this study, we used the near-isogenic wheat lines differing in Al tolerance at a single locus to address the following questions: (1) Does overall growth response of wheat genotypes with contrasting Al tolerance differ in response to elevated [CO_2_] in acidic soil? (2) Is Al-induced malate efflux from wheat roots responsive to elevated [CO_2_]? (3) Is tolerance of wheat to Al changed under elevated [CO_2_]?

## Materials and Methods

### Plants growth

Red loam soils were collected from Qiyang Agricultural Research Station in Hunan Province, China (26°45′N, 111°52′E) with soil pH 5.3. The detailed soil properties used in this study were described by Li et al. (2010). Two wheat genotypes (aluminum-tolerant ET8 and aluminum-sensitive ES8) differing in aluminum tolerance at a single locus were grown in plastic pots (15 cm in diameter × 12 cm in height) filled with 1.6 kg soil. The air-dried soils were sieved (2 mm mesh size) and thoroughly mixed with compound fertilizer (N 16% – P 16% – K 16%). The rate of fertilizer was applied as 0.375 g/kg soil. There were five replicate pots for each treatment, eight plants in each plot. Plants were watered with water to maintain 60–70% of field capacity. Plants were harvested 35 days after growing in ambient or elevated [CO_2_] conditions.

### Open-top chambers

The experiment was carried out in eight octagonal, open-top chambers (OTCs) (1.6 m wide, 4.2 m diameter and 2.4 m high) at the Observation Station on Global Change Biology of the Institute of Zoology, Chinese Academy of Sciences in Xiaotangshan County, Beijing, China (40°11′N, 116°24′E). The current ambient level of CO_2_ (375 ppm) and double the current ambient level of 750 ppm, which is the predicted level in about 100 years (IPCC 2007), were applied continuously in the OTCs. Four blocks were used for CO_2_ treatment. Each block was split into paired OTCs, one with elevated and one with ambient CO_2_.

During 5 weeks of the experiment (9 September to 14 October 2010), 750 ppm CO_2_ concentrations were continuously monitored and controlled by an infrared CO_2_ transmitter (Ventostat 8102, Telaire Company, Goleta, CA) throughout the experiment. CO_2_ concentrations were measured hourly; and the measured CO_2_ concentrations (mean ± SD per day) were 383 ± 26 ppm in ambient CO_2_ chambers versus 769 ± 23 ppm in elevated CO_2_ chambers. Details of the automatic control system for CO_2_ levels and OTCs were described previously (Chen et al. 2005; Sun et al. 2010). The tops of the OTCs were covered with nylon netting to exclude insects. Air temperature was measured three times a day throughout the experiment and did not differ significantly between the two sets of OTCs.

### Measurement of biomass and root length

Roots were scanned with an Epson digital scanner (Expression 10000XL, Epson Inc., Hirooka, Japan) and analyzed with the WinRHIZO/WinFOLIA software (Regent Instruments Inc., Quebec, Canada) to obtain the total length of roots.

### Determination of malate efflux from root apices

Exudation of malic acid from wheat root apices was assayed using the modified method described by Delhaize et al. (1993). Briefly, about 30 root tips (1 cm in length) were excised from the two wheat genotypes grown under conditions of ambient and elevated [CO_2_], and transferred to 5 mL vials containing 1 mL of 0.2 mmol/L CaCl_2_ (pH 4.5) for 2 h after being rinsed with 0.2 mmol/L CaCl_2_ (pH 4.5) for 30 min. To determine the malic acid in solutions, 1 mL of sample was incubated with 1 mL buffer (0.5 mol/L Gly, 0.4 mol/L hydrazine, pH 9.0) and 0.1 mL NAD. After 5 min, the reaction solutions were read under the absorption at 340 nm (the first A_340_). The reaction mixture was incubated for 40–60 min after addition of 5 μL malate dehydrogenase (MDH). The production of NADH led to an increase in A_340_. The change in NADH contents before and after addition of MDH was used to calculate the content of malic acid.

### Determination of intracellular malate content in root apices

To measure intracellular malic acid contents in root apices, 30 root tips were homogenized in liquid N_2_ and extracted using a pestle in 1 mL of ice-cold 0.6 N perchloric acid. The extract was centrifuged at 15,000*g* for 5 min and 0.9 mL of supernatant solution was collected and neutralized with 80 μL of 69% K_2_CO_3_ (W/V). The solution was centrifuged at 15,000*g* for 5 min. Malate content was assayed by mixing 0.5 mL of the supernatant solution and 0.5 mL distilled water as described above.

### Measurement of Al content in root apices

Aluminum contents in root apices were determined following the protocols described previously (Rangel et al. 2007). Briefly, about 20 root tips excised from ET8 and ES8 plants grown in OTC chambers of ambient and elevated [CO_2_] were rinsed three times with 0.2 mmol/L CaCl_2_ (pH 4.5), transferred into 2 mL separate Eppendorf reaction vials and digested in 500 μL ultra-pure HNO_3_ (65%) by overnight shaking on a rotary shaker. The digestion was completed by heating the samples in a water bath at 80°C for 20 min. A quantity of 1.5 mL distilled water was added into the digestion solution after the samples were cooled. All samples were diluted and filtered through 0.45 μm filtering membranes. The Al contents of culture solutions were determined by ICP-OES (ICAP6300; Thermo Scientific, Waltham, MA).

### Statistical analysis

Two-way analysis of variances (ANOVAs) were used to examine effects of genotypes and elevated [CO_2_] on biomass, plant height, root length, malate efflux and malate contents, and Al contents. One-way ANOVA was further used to compare the difference in the above parameters within a genotype between ambient and elevated [CO_2_], and the significant differences between treatments were evaluated by least significance difference multiple range tests (*P* < 0.05) using the SAS statistical software SAS Institute Inc., Cary, NC. Mean values of at least three independent experiments measuring at least five different plants in each experiment and significance were determined by Duncan's multiple range test. Comparison with *P* values of <0.05 were considered significant differently.

## Results

### Effect of elevated CO_2_ on plant biomass

**Figure 1:**
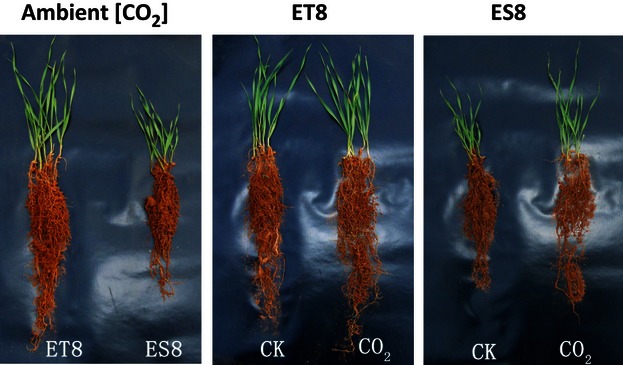
Phenotypes of Al-tolerant ET8 and Al-sensitive ES8 wheat plants grown in acid soils under ambient and elevated [CO_2_] for 5 weeks. CK and CO_2_ mean the wheat seedlings grown under ambient and elevated [CO_2_] conditions, respectively.

**Figure 2:**
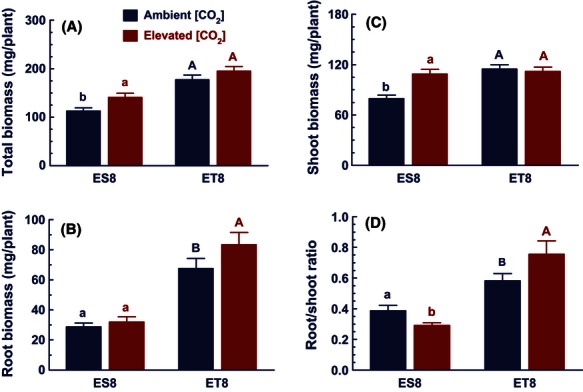
Effect of elevated [CO_2_] on total biomass (A), root biomass (B), shoot biomass (C), and root/shoot ratio of Al-tolerant ET8 and Al-sensitive ES8 plants. Data are mean ± SE with at least for five seedlings for each treatment. Different letters within each genotype mean statistically significant at *P* < 0.05.

In this study, we used the near-isogenic wheat lines that differ in Al^3+^ tolerance at a single locus to study the responses of wheat to elevated [CO_2_] by growing them in acidic soils. Two-way ANOVA analyses revealed that both genotype and elevated [CO_2_] had impacts on total biomass, but no interactive effect of elevated [CO_2_] and genotype on total biomass was observed (elevated [CO_2_]: *F* = 57.24, *P* < 0.0001; Genotype: *F* = 6.52, *P* = 0.0189; [CO_2_] × genotype: *F* = 0.01, *P* = 0.937). Aluminum-tolerant ET8 wheat had greater biomass than ES8 wheat when grown in acidic soils under conditions of ambient [CO_2_] (Fig. 1). There was a significant increase in overall biomass of Al-sensitive ES8 wheat in response to elevated [CO_2_] (Fig. 2A). In contrast, exposure of Al-tolerant ET8 wheat to the same elevated [CO_2_] regimes led to an insignificant increase in its overall biomass (Fig. 2A). We further investigated the response of shoot and root biomass to the elevated [CO_2_] for the two wheat genotypes. Similar to total biomass, both elevated [CO_2_] and genotype had significant effect on root biomass, and there was no interactive effect of elevated [CO_2_] and genotype on root biomass (elevated [CO_2_]: *F* = 58.62, *P* < 0.0001; Genotype: *F* = 5.56, *P* = 0.0263; elevated [CO_2_] × genotype: *F* = 1.42, *P* = 0.2469). Under conditions of ambient [CO_2_], root biomass of ET8 was significantly higher than that of ES8, whereas no difference in shoot biomass between ES8 and ET8 after exposure to the elevated [CO_2_] was observed (Fig. 2B). Shoot biomass was significantly affected by both elevated [CO_2_] and genotype, an interactive effect of elevated [CO_2_] and genotype on shoot biomass was observed (elevated [CO_2_]: *F* = 25.13, *P* < 0.0001; genotype: *F* = 9.00, *P* = 0.0071; elevated [CO_2_] × genotype: *F* = 7.53, *P* = 0.0125). Like the overall biomass, shoot biomass of ES8 was significantly increased in response to the elevation of [CO_2_], whereas shoot biomass of ET8 remained relatively unchanged in response to elevation of [CO_2_] (Fig. 2C). The contrasting response of shoot and root biomass between ES8 and ET8 wheat plants to elevated [CO_2_] led to a significant decrease and increase in root/shoot ratio for ES8 and ET8 after exposure to elevated [CO_2_] (Fig. 2D).

### Effect of elevated CO_2_ on plant height and root length

**Figure 3:**
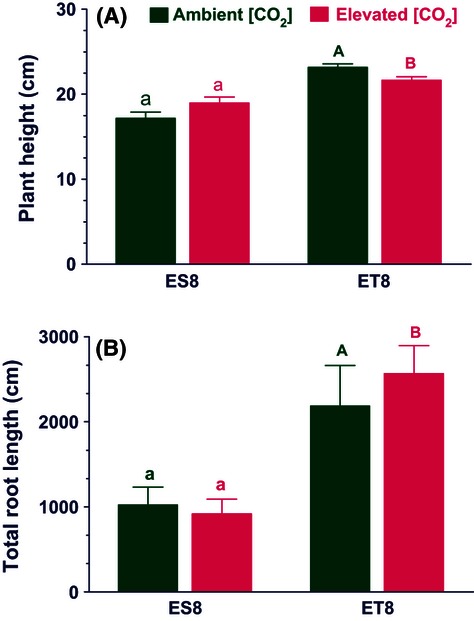
Effect of elevated [CO_2_] on plant height (A) and total root length (B) of Al-tolerant ET8 and Al-sensitive ES8 plants. Data are mean ± SE with at least for five seedlings for each treatment. Different letters within each genotype mean statistically significant at *P* < 0.05.

In addition to biomass, we also measured the effect of elevated [CO_2_] on plant height and root length of the two wheat genotypes. Elevated [CO_2_], but not genotype, had a significant effect on plant height, and no interactive effect of elevated [CO_2_] and genotype on plant height was observed (elevated [CO_2_]: *F* = 37.94, *P* < 0.0001; genotype: *F* = 0.72, *P* = 0.4088; elevated [CO_2_] × genotype: *F* = 1.42, *P* = 0.2503). As shown in Figure 3A, ET8 had higher height than ES8 under ambient [CO_2_] conditions, and exposure of ES8 to the elevated [CO_2_] did not affect its height. Both elevated [CO_2_] and genotype had a significant effect on total root length, but the two factors had no interactive effect (elevated [CO_2_]: *F* = 26.12, *P* < 0.0001; genotype: *F* = 6.45, *P* = 0.0125; elevated [CO_2_] × genotype: *F* = 0.64, *P* = 0.4328). The higher height of ET8 than ES8 under ambient [CO_2_] conditions may be accounted for by that ES8 was more sensitive to Al than ET8, leading to malfunction of root in ES8 in terms of water and nutrient acquisition due to inhibition of root growth by Al. The observation that the overall root length of ET8 was 2.1-fold longer than that of ES8 under ambient [CO_2_] conditions (Fig. 3B) is consistent with this explanation. Exposure of ET8 and ES8 to the elevated [CO_2_] led to different effect on their overall root length such that root length of ET8 was significantly increased by the elevated [CO_2_], whereas root length of ES8 was not significantly affected by the elevated [CO_2_] (Fig. 3B). These results highlight that wheat genotypes with contrasting Al tolerance exhibit contrasting strategy to allocation of carbon in response to the elevated [CO_2_], that is, Al-tolerant wheat ET8 allocated C to roots, whereas Al-sensitive ES8 wheat allocated C to shoot in response to [CO_2_] elevation.

### Effect of elevated [CO_2_] on exudation of malate and intracellular malate contents

**Figure 4:**
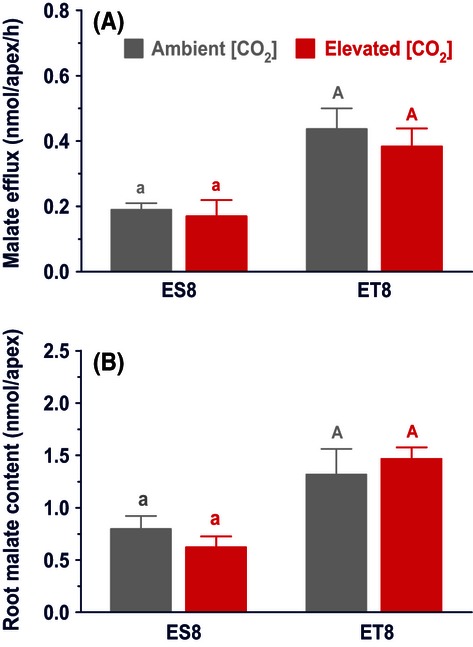
Effect of elevated [CO_2_] on malate exudation rate from root apices (A) and intracellular malate contents in root apices (B) of Al-tolerant ET8 and Al-sensitive ES8 plants. Data are mean ± SE with three replicates for each treatment. Different letters within each genotype mean statistically significant at *P* < 0.05.

To test whether the two wheat genotypes differed in their capacity to release malate grown in acidic soils under ambient and elevated [CO_2_] conditions, we compared malate efflux from root apices of ET8 and ES8 grown under the two [CO_2_] regimes. No significant effects of elevated [CO_2_] and genotype as well as their interaction on malate efflux from root apices were found (elevated [CO_2_]: *F* = 0.05, *P* = 0.8205; genotype: *F* = 1.23, *P* = 0.3005; elevated [CO_2_] × genotype: *F* = 0.87, *P* = 0.3784). However, the one-way ANOVA analysis showed that efflux rate of malate from root apices of ET8 was significantly greater than that from ES8 in ambient [CO_2_] conditions (Fig. 4A). This result confirms that Al-tolerant ET8 has greater capacity to adapt to acidic soils in which Al toxicity dominates than Al-sensitive ES8 wheat. Exposure of ET8 and ES8 plants to elevated [CO_2_] had no impact on their malate efflux such that malate efflux from ET8 root apices was still much greater than from ES8 root apices. In addition to malate efflux rate, we also measured intracellular malate contents in root apices of the two wheat genotypes under ambient and elevated [CO_2_]. Genotype, but not elevated [CO_2_], had significant effect on malate contents in roots, and there was no interactive effect of the two factors on root malate contents (elevated [CO_2_]: *F* = 0.01, *P* = 0.9373; genotype: *F* = 18.80, *P* = 0.0025; elevated [CO_2_] × genotype: *F* = 1.06, *P* = 0.3333). There was no significant difference in malate contents in root apices between ET8 and ES8 under ambient conditions. A marginal decrease and increase in malate content in ES8 and ET8 root apices was found in response to exposure to elevated [CO_2_] (Fig. 4B). In contrast to ambient [CO_2_] conditions, malate contents in root apices of ET8 were significantly higher than those of ES8 when they were grown under conditions of elevated [CO_2_].

### Effect of elevated [CO_2_] on Al contents in root tips

**Figure 5:**
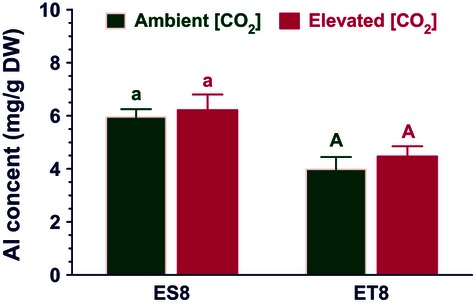
Effect of elevated [CO_2_] on Al content in root apices of Al-tolerant ET8 and Al-sensitive ES8 plants. Data are mean ± SE with three replicates. Different letters within each genotype mean statistically significant at *P* < 0.05.

The greater amounts of malate released from root apices of ET8 would reduce Al contents in their root apices by forming Al-malate complex. To test this possibility, we measured Al contents in root apices in the two wheat genotypes grown under the two [CO_2_] regimes. Elevated [CO_2_] had no significant effect on Al contents in root apices (elevated [CO_2_]: *F* = 1.52, *P* = 0.2357), whereas genotype significantly impacted Al contents in root apices (genotype: *F* = 21.90, *P* = 0.0003). No interactive effect of elevated [CO_2_] and genotype on Al contents in root apices was observed (elevated [CO_2_] × genotype: *F* = 0.40, *P* = 0.5347). In addition, Al contents in ES8 root apices were significantly higher than in ET8 root apices (*P* = 0.0078) under ambient [CO_2_]. No significant differences were found in Al contents in root apices for both ET8 and ES8 between ambient and elevated [CO_2_] (Fig. 5). Although Al contents in root apices of both wheat genotypes under ambient [CO_2_] were not significantly different from those under elevated [CO_2_], Al contents in root apices of ES8 were significantly higher (*P* = 0.0375) than in root apices of ET8 under elevated [CO_2_], confirming the greater tolerance of ET8 plants to Al toxicity than that of ES8 plants.

## Discussion

Soil acidification due to excessive application of N fertilizers has been a serious problem worldwide. Atmospheric [CO_2_] concentrations have continuously risen since 1800 (IPCC 2007). The elevated [CO_2_] has profound impact on vegetation, ecosystem function, and food supply. Despite of extensive research on the effect of elevated [CO_2_] on plant growth at multilevels, no detailed study has been conducted to evaluate growth response of plants grown on acid soils to elevated [CO_2_]. In this study, we investigated the effect of elevated [CO_2_] on two wheat genotypes differing in tolerance to Al by growing them in acidic soils. Our results showed that the two wheat genotypes differed in their growth patterns in response to elevated [CO_2_], such that elevated [CO_2_] enhanced root and shoot growth of Al-tolerant ET8 and Al-sensitive ES8 seedlings, leading to increase and decrease in root/shoot ratio in the two wheat genotypes, respectively. We further demonstrated that there was no significant difference in malate efflux from root apices of Al-tolerant ET8 plants grown under ambient and elevated [CO_2_] conditions when expressed on the basis of root apices. These findings have important implications for our understanding how plants respond to elevated [CO_2_] grown in unfavorable edaphic conditions in general and in acidic soils in particular.

There have been numerous studies on the effect of elevated [CO_2_] on biomass of different plant species in the literature with varying responses of biomass to elevated [CO_2_] under varying growth conditions, such as salinity, low and high temperature, drought and nutrient deficiencies in general, and nitrogen deficiency in particular (cf. Poorter and Pérez-Soba 2001; Lenka and Lal 2012). In general, a decrease in nutrient availability dampens the relative growth response of plants to [CO_2_]. For instance, Haase et al. (2008) reported that elevated [CO_2_]-induced increases in both shoot and root biomass are abolished by Fe deficiency in barley seedlings grown in both hydroponic and soils. Jin et al. (2009) showed that tomato seedlings grown in Fe-deficient medium can increase Fe mobilization by up-regulating ferric reductase activity in response to elevated [CO_2_], thus conferring Fe-deficient seedlings greater increase in biomass of both shoot and root than Fe-sufficient seedlings under elevated [CO_2_]. In addition to Fe, elevated [CO_2_] has been reported to enhance both root and shoot growth of chickpea and wheat grown in phosphate-sufficient vertosol soils, whereas the stimulatory effect of elevated [CO_2_] on shoot and root growth of chickpea and wheat was abolished when they were grown in phosphate-deficient soils (Jin et al. 2012). In a recent study, Niu et al. (2013) reported that elevated [CO_2_] has different effect on growth of Arabidopsis seedlings in P-deficient medium in the presence of NO_3_^−^ and NH_4_^+^. The differential response of root and shoot growth to elevated [CO_2_] for the NO_3_^−^ and NH_4_^+^-fed plants led to an increase in root/shoot ratio for NO_3_^−^-fed plants exclusively. The authors also showed that exposure of P-deficient Arabidopsis seedlings to elevated [CO_2_] led to up-regulation of genes involved in uptake, translocation, and mobilization of P in the NO_3_^−^-fed, but not NH_4_^+^-fed plants (Niu et al. 2013). These findings, together with our results reported in this study, highlight the important roles of soil mineral composition in controlling plant growth under short-term elevated [CO_2_].

There are reports of the effect of elevated [CO_2_] on plant growth grown in acid soils (Hagedorn et al. 2002). However, these studies did not specifically focus on toxic effect of Al^3+^ ions. For instance, Hagedorn et al. (2002) investigated the effects of elevated [CO_2_] on nutrient availability in young beach and spruce plants grown in acidic loam (pH 4.1–4.2) and calcareous sand (pH 7.1–7.2) soils. Their findings that the response of nutrient accumulation in the two species to elevated [CO_2_] differs substantially may partly be accounted for by the potential effect of Al on nutrient accumulation in the acidic soils and/or the difference in tolerance of the two species to Al toxicity. In this context, it has been well established that uptake and transport of several mineral nutrients (K^+^, Mg^2+^, Ca^2+^) are suppressed by Al (Rengel and Zhang 2003).

In this study, we demonstrate that wheat plants differing in their tolerance to Al showed contrasting response to elevated [CO_2_] when grown in acidic soils in terms of shoot and root growth. At the whole plant level, elevated [CO_2_] enhanced biomass in Al-sensitive ES8 wheat, but no significant changes in biomass of Al-tolerant ET8 plants were observed. These findings suggest that wheat genotypes with contrasting Al tolerance exhibit contrasting strategy to allocation of carbon in response to short-term elevated [CO_2_], that is, Al-tolerant wheat ET8 allocated C to roots, whereas Al-sensitive ES8 wheat allocated C to shoot in response to [CO_2_] elevation. These observations may be accounted for by that toxic Al restricts root growth in Al-sensitive ES8 plants, and the elevated [CO_2_]-induced increase in photoassimilate has to be allocated to above-ground tissues, thus leading to the stimulation of shoot growth. These findings are of importance for our understanding of response of plant biomass to elevated [CO_2_], such that soil nutrient status and toxic metals as well as species and/or genotypes with different capacities to tolerate the soil conditions should be included in explanation of elevated [CO_2_]-dependent changes in biomass. Moreover, our results may also account for the varying effects of elevated [CO_2_] on plant growth reported in the literature as these studies hardly take into account the differences in traits of plant species on tolerance to soil toxic metals and soil mineral status.

Another interesting observation in this study is that elevated [CO_2_] had no impact on malate efflux from ET8 root apices (cf. Fig. 4A), suggesting that malate-dependent Al tolerance mechanism is not affected by elevated [CO_2_]. The observation that Al content in root apices of both ET8 and ES8 grown in ambient [CO_2_] did not significantly differ from those grown in elevated [CO_2_] (Fig. 5) is consistent with the effect of elevated [CO_2_] on malate exudation. Watt and Evan (1999) reported that elevated [CO_2_] has no impact on exudation of citrate from cluster roots of white lupin. A similar result that citrate exudation from cluster roots of white lupin is not affected by elevated [CO_2_] has also been reported by Wasaki et al. (2005). Like our results, citrate exudate rate was determined as per root (Wasaki et al. 2005) or root length (Watt and Evan 1999). Elevated [CO_2_] can increase the cluster root number (Wasaki et al. 2005). Therefore, although citrate exudation rate per root is not affected by elevated [CO_2_], the overall citrate exudation from cluster roots is likely to be enhanced under elevated [CO_2_] due to enhancement of root systems. In our study, we found elevated [CO_2_] stimulated root growth of ET8 plants as evidenced by increases in root biomass (Fig. 2). It is conceivable that more lateral roots may be developed in ET8 plants under elevated [CO_2_]. This would lead to a more root tips and thus the malate efflux would be enhanced though malate efflux rate despite malate efflux rate of per root apices was not affected by elevated [CO_2_]. In addition, our results that root biomass of ET8 plants was significantly enhanced by elevated [CO_2_] (Figs. 2) and that the overall root length of ET8 plants was not significantly affected under elevated [CO_2_] may also imply that more roots due to stimulation of lateral root initiation occur when ET8 plants were exposed to elevated [CO_2_]. Elevated [CO_2_] enhanced overall biomass of ES8 plants by 24% (Fig. 2A), whereas it marginally enhanced ET8 plants by only 10% (Fig. 2). Assuming that elevated [CO_2_] has similar effect on the overall photosynthesis in ET8 and ES8 plants, the difference in the [CO_2_]-induced biomass stimulation between ET8 and ES8 may be accounted for by the overall C exudation via malate efflux from roots. Elevated [CO_2_] stimulated total root length in ET8 plants by 17% (Fig. 2). The increase in the root length may partly result from initiation of lateral roots and their growth, thus leading to more malate efflux under elevated [CO_2_]. In white lupin, elevated [CO_2_] increased cluster root number by 50% (Wasaki et al. 2005). If a similar stimulatory effect of elevated [CO_2_] on lateral root number occurs in wheat roots in our study, it may suggest that elevated [CO_2_] would enhance overall malate efflux from wheat root tips due to increases in lateral root tips although malate effect on the basis of individual root tips was not affected by elevated [CO_2_]. Phillips et al. (2009) showed that root exudation from loblolly pine (*Pinus taed*a) is stimulated by elevated CO_2_ and that the stimulatory effect is dependent on N status in soils, such that the elevated CO_2_-induced root exudation is more pronounced under low N supply than under high N supply. Therefore, response of root exudation to elevated CO_2_ is closely related to nutrient availability in soils. However, previous studies mainly focus on the effect of elevated [CO_2_] on root exudation under varying supply of those mineral nutrients essential for plant growth and development. Our study is the first one, to the best knowledge of authors, to evaluate the effect of elevated [CO_2_] on root exudation in the presence of toxic Al, which occurs predominantly in acid soils.

In summary, we demonstrate that wheat genotypes with contrasting tolerance to Al toxicity differed markedly in their response to elevated [CO_2_] when grown in acid soils, such that an increase in root biomass was found in Al-tolerant ET8 wheat under elevated [CO_2_], whereas an increase in shoot biomass was observed in Al-sensitive ES8 wheat under the same elevated [CO_2_] regime. In addition, Al-induced malate efflux from wheat root tips was not affected by elevated [CO_2_], suggesting that tolerance of wheat plants to Al is not altered under elevated [CO_2_].
